# *Streptococcus equi* subspecies *equi* pyelonephritis in a pony

**DOI:** 10.1128/asmcr.00240-25

**Published:** 2026-05-14

**Authors:** Maho Okumura, Louise Southwood, Nathan Helgert, Cristobal Navas de Solis, Cassandra Mahoney, Donna J. Kelly

**Affiliations:** 1 University of Pennsylvania School of Veterinary Medicine 6572, Philadelphia, Pennsylvania, USA; 2 New Bolton Center, University of Pennsylvania School of Veterinary Medicine 189482, Kennett Square, Pennsylvania, USA; 3 Farmview Veterinary Services, Lincoln University 4558, Philadelphia, Pennsylvania, USA; Pattern Bioscience, Austin, Texas, USA

**Keywords:** metastatic strangles, pyelonephritis, strangles, *Streptococcus equi *subspecies *equi*

## Abstract

**Background:**

*Streptococcus equi* subspecies *equi* (*SEE*) causes a highly contagious disease colloquially known as “strangles” in equids. This disease is typically characterized by pyrexia, mucopurulent nasal discharge, and abscessation of the submandibular and retropharyngeal lymph nodes. Although typically localized to the upper respiratory tract, the infection can disseminate via lymphatic or hematogenous routes, leading to disseminated strangles involving peripheral lymph nodes and thoracic and abdominal organs.

**Case Summary:**

A 21-year-old Welsh Pony cross gelding presented for urine dribbling and presumptive cystolithiasis due to urine retention and bladder distention. Urinary catheterization and urethrocystoscopy revealed a large volume of hematuria, no urinary calculi, and an abnormal ureteral opening. Imaging revealed nonspecific renal abnormalities consistent with chronic kidney disease, with secondary acute kidney injury, and a mass near the aortic bifurcation. Aerobic urine culture yielded *SEE* identified via matrix-assisted laser desorption/ionization time-of-flight mass spectrometry with a score >2.0 and confirmed by PCR. The pony was euthanized due to poor prognosis, and autopsy results revealed pituitary pars intermedia hyperplasia and microadenoma and neutrophilic, hemorrhagic, and necrotizing pyelonephritis. The pyelonephritis is likely due to hematogenous or lymphatic dissemination of the organism to the kidney. We speculate that immunosuppression related to pars pituitary intermedia dysfunction may have predisposed this animal to this atypical manifestation.

**Conclusion:**

There are limited reports of metastatic strangles presenting as pyelonephritis in equids. This case highlights the potential for a common equine pathogen to manifest as an unexpected presentation and underscores the need to consider strangles in the differential diagnosis of equine renal disease.

## INTRODUCTION

*Streptococcus equi* subspecies *equi* (*SEE*) are gram-positive, beta-hemolytic cocci in Lancefield group C that cause a highly contagious upper respiratory tract infection known as “strangles” in horses (1). Transmission occurs via direct or indirect contact with infectious material. The bacterium attaches to epithelial cells in the tonsillar crypts and subsequently invades adjacent mandibular and pharyngeal lymphoid tissues (2, 3). Clinically, strangles is characterized by acute pyrexia, mucopurulent nasal discharge, and abscessation of the submandibular and retropharyngeal lymph nodes, which may drain into the guttural pouches (1, 2). Complications include a persistent carrier state, disseminated strangles, and purpura hemorrhagica (2). Disseminated spread occurs via hematogenous or lymphatic migration of the organism to distant organs (4). Because of this variability, strangles presents a diagnostic challenge. Although the urinary tract has been cited as a possible site of metastatic infection, confirmed reports of pyelonephritis are limited.

## CASE PRESENTATION

A 21-year-old Welsh Pony cross gelding (270 kg) presented to the University of Pennsylvania New Bolton Center for urine dribbling and presumptive cystolithiasis. Three weeks prior to presentation, he developed pyrexia (39.2°C; reference range 37.3°C–38.2°C) and was initially treated with IV flunixin meglumine (1.1 mg/kg as needed) and oxytetracycline (6.6 mg/kg IV q24h for 5–7 days), after which he was transitioned to doxycycline (10 mg/kg PO q12h for 7 days). On the morning of presentation prior to referral, he had elevated serum amyloid A (1,833 μg/mL), fibrinogen (758 mg/dL), plasma creatinine (7.6 mg/dL), blood urea nitrogen (BUN) (97 mg/dL), potassium (6.6 mmol/L), and total plasma protein (9.3 g/dL). Neutrophilic leukocytosis (18,000 leukocytes/μL and 15,420 segmented neutrophils/μL) was present. The gelding was current on core vaccinations in accordance with the American Association of Equine Practitioners guidelines, had a negative equine infectious anemia agar gel immunodiffusion test, and had not received any recent vaccinations (5).

The gelding was quiet and alert on presentation, with a stiff gait. His vital parameters were within normal limits (HR 40 bpm, RR 20 bpm, T 37.7°C). He displayed an elevated tail carriage and was observed straining and dribbling urine. Mucous membranes were pink and moist, with a capillary refill time of <2 seconds. Borborygmi were present in all four abdominal quadrants. Mildly increased digital pulses were noted in all limbs. Transrectal pelvic examination revealed a markedly distended and taut bladder. After sedation with acepromazine, xylazine, and butorphanol, urinary catheterization was performed following routine aseptic preparation of the penis and prepuce using sterile gloves, catheter, and lubricant. Initially, dark red, highly viscous urine was obtained, followed by approximately 6 L of clearer urine with a hemorrhagic tinge.

Urinalysis performed on intake revealed isosthenuria (specific gravity 1.013), numerous white blood cells and leukocyte casts, 4+ blood, and 1+ cocci. A urine sample was submitted for aerobic and anaerobic culture. The gelding’s complete blood count and chemistry panel were within normal limits other than markedly increased total solids, potassium, creatinine, and BUN, indicative of renal disease (Table 1).

**TABLE 1:** Relevant point-of-care bloodwork results^*a*^

Parameter	Value
Packed cell volume	36% (31.1–50.0%)
Total solids	9.8 g/dL (4.6–6.9 g/dL)
Sodium	136.1 mmol/L (132–141 mmol/L)
Potassium	5.1 mmol/L (2.7–3.9 mmol/L)
Chloride	102.7 mmol/L (94–102 mmol/L)
Ionized magnesium	0.93 mg/dL (1.6–2.5 mg/dL)
Total carbon dioxide	30 mmol/L (24–31 mmol/L)
Lactate	1.1 mmol/L (<1 mmol/L)
Creatinine	4.3 mg/dL (0.6–1.8 mg/dL)
Blood urea nitrogen	114 mg/dL (8–27 mg/dL)

^*a*^ Reference ranges shown in parentheses.

Cystoscopy revealed a normal urethra and bladder. The right ureteral opening was abnormally hyperemic and mildly thickened. The left ureteral opening was unremarkable. Transcutaneous and transrectal ultrasonography demonstrated a markedly enlarged right kidney with poor corticomedullary distinction, a dilated renal pelvis (1.5 cm), and triangular echoic areas in the cortex consistent with renal infarcts. The right ureter was subjectively thick, corrugated, and distended. The left kidney was notably small with almost complete loss of normal architecture. A mass was identified cranial to the right side of the aortic bifurcation that included a hypoechoic striated root of the mesentery and a hypo- to heteroechoic mass.

Based on these findings, the gelding was diagnosed with chronic kidney disease (CKD) with secondary acute kidney injury (AKI) consistent with pyelonephritis, glomerulonephritis, or other toxic or inflammatory insults. Differential diagnoses for the mass included abscess, granuloma, lymphadenopathy, or neoplasia.

The gelding was given isotonic intravenous fluid therapy (0.5 L/hour), and urine output was subjectively monitored. Given the poor prognosis based on severe renal disease, euthanasia was elected. The body was submitted for an autopsy to the Pennsylvania Diagnostic Laboratory System Mammalian Pathology Laboratory.

**Fig 1:**
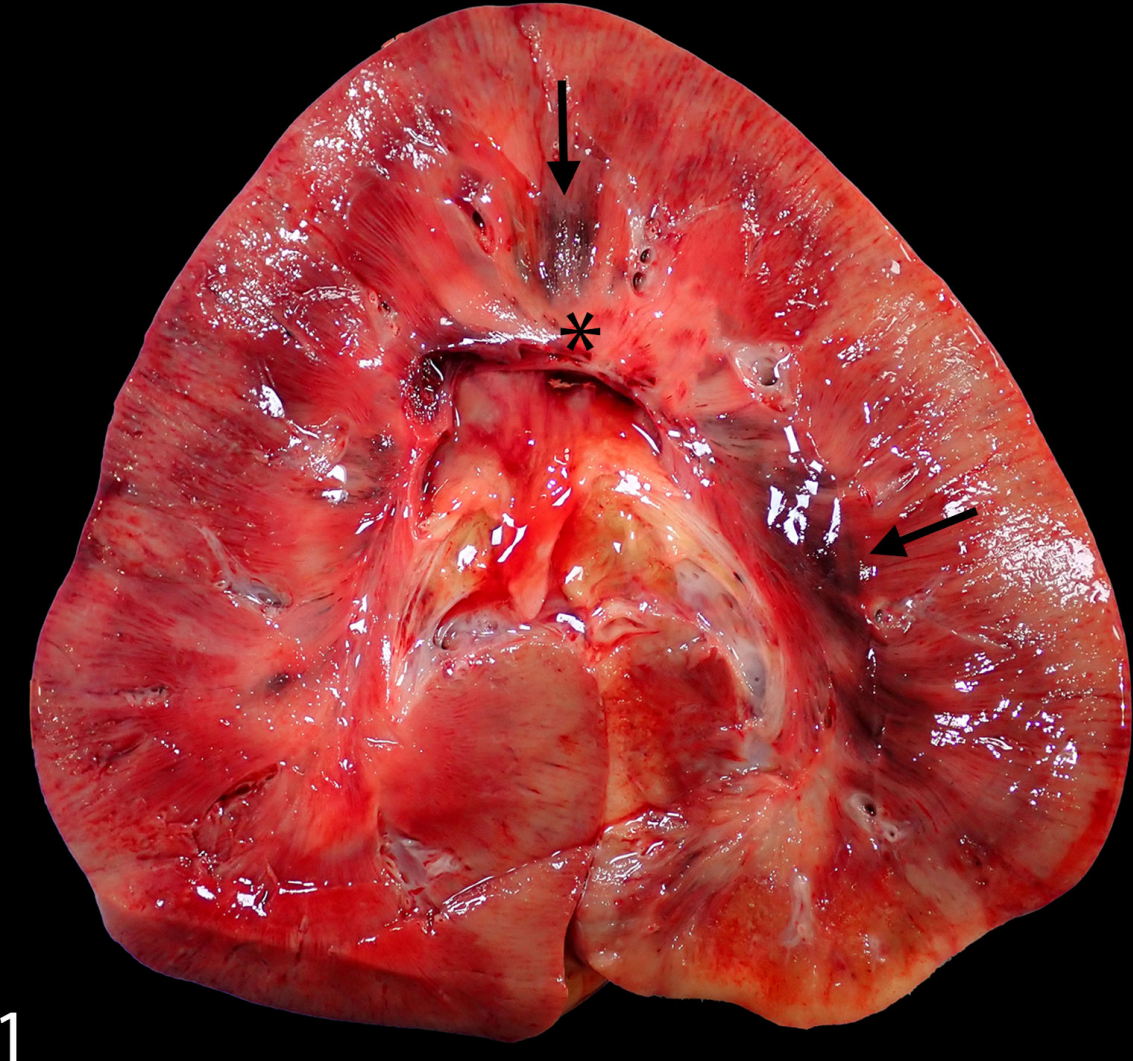
Right kidney. Throughout the medulla and occasionally extending into the cortex are multiple areas of hemorrhage (arrows). The renal crest is irregular with areas of hemorrhage (asterisk).

At postmortem examination, the right inguinal and iliac lymph nodes were markedly enlarged to approximately fourfold the contralateral lymph nodes and were filled with abundant purulent exudate when cut. The right kidney was approximately 1.5-fold larger than expected (21 × 20 cm), and on cut section, contained a moderate amount of cloudy to opaque yellow-pink fluid in the renal pelvis and ureter. Throughout the renal medulla, cortex, and renal crest were multiple areas of hemorrhage (Fig. 1). The left kidney was approximately half of the expected size (9 × 13 cm) and was firm and white-tan with scattered pink-purple nodules ranging from 0.5 to 2 cm in diameter. The urinary bladder contained abundant opaque to flocculent red-pink fluid and a moderate amount of gritty yellow material. The pituitary gland was markedly enlarged with a firm, tan nodule in the pars intermedia.

**Fig 2:**
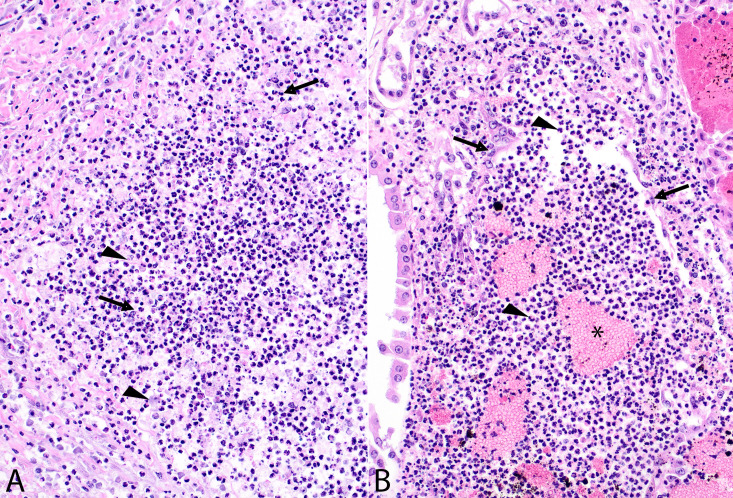
Histopathological findings. (**A**) Lymph node, inguinal. The lymph node is infiltrated by abundant neutrophils (arrows) and macrophages (arrowheads). Original magnification 200×. Hematoxylin and eosin. (**B**) Kidney. Renal tubules are lined by attenuated epithelial cells (arrows) and are filled with abundant neutrophils (arrowheads) and erythrocytes (asterisk). Original magnification 200×. Hematoxylin and eosin.

Tissues fixed in 10% neutral buffered formalin were routinely processed, embedded in paraffin, sectioned, and stained with hematoxylin and eosin and/or McDonald Gram stain. The right inguinal and iliac lymph nodes were variably necrotic with a marked infiltrate of neutrophils (Fig. 2). The right kidney had marked neutrophilic, hemorrhagic, and necrotizing pyelonephritis. The left kidney contained severe widespread cystic renal tubular degeneration, glomerulosclerosis, interstitial fibrosis, and interstitial lymphoplasmacytic inflammation, consistent with chronic infarction or previous bouts of pyelonephritis. No microorganisms were noted. The pituitary pars intermedia was hyperplastic with a microadenoma. No other significant histologic abnormalities were identified.

The urine sample collected at intake was streaked on tryptic soy agar with 5% sheep blood (BAP), MacConkey agar, and Columbia agar with colistin and nalidixic acid (Remel, Lenexa, KS) and incubated for 24–48 hours at 35°C–37°C in 7% CO_2_ for aerobic culture. Anaerobic culture was performed on Brucella agar with 5% sheep blood, hemin, and vitamin K, phenylethyl alcohol agar, and Bacteroides bile esculin and laked kanamycin vancomycin bi-plate (Remel, Lenexa, KS), incubated for 7 days at 35°C–37°C in anaerobic conditions. No anaerobic growth was observed. Heavy growth of beta-hemolytic, 0.1 cm–0.3 cm diameter mucoid colonies (>100,000 cfu/mL) was observed on BAP and Columbia agar plates within 24 hours. No growth occurred on the MacConkey agar. Colonies were identified using matrix-assisted laser desorption/ionization time-of-flight mass spectrometry (Sirius 1 Biotyper, Bruker, Billerica, MA), confirming *Streptococcus equi* subsp. *equi* (score >2.0). Antimicrobial susceptibility testing was performed; however, results are not presented as they did not influence clinical decision-making due to euthanasia shortly after presentation, and the susceptibility profile was consistent with expected patterns for *SEE*. Colonies were submitted to the University of California, Davis Veterinary Diagnostic RT-PCR Research and Diagnostics Core Facility for confirmatory molecular testing. RT-PCR targeting the *SeM* and *seq2190* genes confirmed *SEE*. Additional PCR assays differentiating *S. equi* subsp. *zooepidemicus* (targeting the *szp* gene) and the live vaccine strain (targeting the *SeM* gene variant) verified infection with a wild-type strain of *SEE*.

## DISCUSSION

There are limited reports of *SEE* pyelonephritis in equids, and to our knowledge, this is the first reported case with presumptive pituitary pars intermedia dysfunction (PPID) (6, 7). Rare mentions of *SEE* pyelonephritis exist in the literature, where renal or abdominal involvement by *SEE* has been observed in the context of broader case series. Laus et al. described *SEE* abdominal lesions with incidental renal involvement, although pyelonephritis was not the primary focus of the report (7). Typical presentations of metastatic strangles include suppurative bronchopneumonia and abscessation in the mesentery, liver, spleen, kidneys, and brain; however, metastatic abscessation is uncommon, reported in approximately 2%–28% of outbreaks (4, 8, 9). Diagnosis of disseminated strangles may be difficult, so a history of exposure to *SEE*, intermittent low-grade fevers, responsiveness to penicillin, and increased *SeM*-specific antibody titers are supportive of diagnosis (4).

The pathogenesis of urinary involvement likely reflects hematogenous or lymphatic dissemination from an unrecognized previous infection, likely during the period of pyrexia 3 weeks prior to presentation. *SeM* and related virulence factors confer resistance to phagocytosis, complement-mediated killing, and host immune clearance, allowing persistence within abscesses (1, 4). Once systemic, *SEE* localizes in tissues with preexisting disease. In this gelding, the pituitary changes identified at necropsy are commonly associated with PPID, a plausible predisposing factor. PPID-associated immune dysregulation may predispose affected horses to opportunistic infection (10). Age-related renal degeneration and altered vascular integrity may have further facilitated bacterial seeding within the renal parenchyma.

There are several limitations to this gelding’s diagnosis. It is not possible to determine whether the gelding was a subclinical carrier or experienced reinfection. Although common causes of equine CKD include unresolved injuries from toxins, infectious agents, and immune-mediated inflammation, the cause of CKD could not be definitively established. Furthermore, pre-referral antimicrobial therapy with oxytetracycline or another nephrotoxic factor may have contributed to the AKI. Blood cultures were not performed, as they are not routinely pursued without suspicion of septicemia, and euthanasia was elected shortly after presentation.

In summary, the findings of this case suggest that host immune status, rather than pathogen tropism alone, may determine the pattern of *SEE* dissemination. Clinicians should recognize that *SEE* infections, regardless of presentation, warrant definitive diagnostic confirmation at the subspecies level and compliance with regulatory reporting requirements.
